# Association of exercise and race/ethnicity with atypical femur fracture

**DOI:** 10.1093/jbmrpl/ziaf071

**Published:** 2025-04-23

**Authors:** Annette L Adams, Margo Sidell, Alec D Gilfillan, Denison S Ryan, Jörg Schilcher, Dennis M Black

**Affiliations:** Department of Research and Evaluation, Kaiser Permanente Southern California, Pasadena, CA 91101, United States; Department of Research and Evaluation, Kaiser Permanente Southern California, Pasadena, CA 91101, United States; Department of Research and Evaluation, Kaiser Permanente Southern California, Pasadena, CA 91101, United States; Department of Research and Evaluation, Kaiser Permanente Southern California, Pasadena, CA 91101, United States; Department of Biomedical and Clinical Sciences and Department of Orthopaedics, Linköping University, Linköping, Sweden; Department of Epidemiology and Biostatistics, University of California San Francisco, San Francisco, CA 94143, United States

**Keywords:** atypical femur fractures, fractures, osteoporosis treatment, bisphosphonates, activity levels, weight-bearing exercise

## Abstract

Atypical femur fractures (AFF) resemble stress fractures. Stress fractures can result from repetitive stresses like weight-bearing exercise and femoral geometry may influence where stress fractures manifest along the bone. Atypical femur fractures risk is demonstrably greater among Asian women compared to Caucasian women, with differences in femoral geometry hypothesized to contribute to elevated risk. Using a previously established cohort of older adult women, all with bisphosphonate (BP) use sometime during the study period (2010-2017) and some with AFF, we linked self-reported information on weekly exercise to enable evaluation of whether the average weekly minutes of exercise differed by race/ethnicity and whether accounting for any differences explains the increased AFF risk seen among Asian women. Data came from the longitudinal electronic health record of Kaiser Permanente Southern California. Atypical femur fractures were identified by expert review and adjudication of femur fracture radiographs. Race/ethnicity was categorized as White, Asian, and Other. Average weekly minutes of exercise were expressed in units of 15-min blocks. Bivariable and multivariable Poisson regression models were used to estimate incidence rate ratios (IRR) and 95% CI for the associations between exercise, race/ethnicity, and AFF. Exercise levels for all three race/ethnicity categories were different from each other at *p* < .001. After adjusting for age, cumulative duration of BP use, and recency of BP use, the IRR for AFF risk in Asian women (compared to White women) was 4.96 (CI, 3.72-6.61). Adding exercise level to this same model did not change the estimate for Asian women meaningfully (IRR 5.03, CI, 3.77-6.71). Thus, the observed race/ethnicity variation in exercise levels does not appear to contribute to the elevated risk of AFF in Asian women.

## Introduction

Atypical femur fractures (AFF) resemble stress fractures, which result from repetitive stresses and femoral geometry influences where stress fractures manifest along the bone. Atypical femur fractures risk is demonstrably greater among Asian women compared to Caucasian women,^1–5^ with differences in femoral geometry^3^^,^^6–10^ hypothesized to contribute to elevated risk. Differences in repetitive stresses in the form of weight-bearing exercises may interact with geometric variations in femoral geometry, resulting in the observed race/ethnicity-associated AFF incidence. We leveraged novel measures of moderate-to-strenuous activity levels to evaluate the association with race/ethnicity within a previously described cohort of women treated with bisphosphonates (BPs), a portion of whom developed AFFs.^11^

## Materials and methods

This retrospective cohort study included female members of Kaiser Permanente Southern California (KPSC), a large integrated healthcare system in the United States, aged ≥50 yr, and treated with BP for osteoporosis or osteopenia anytime during 2010-2017. The first day of membership between January 1, 2010 and December 31, 2017 was denoted as the index date. Individuals were followed from the index date until the earliest of (1) occurrence of an AFF, (2) death or health plan disenrollment, or (3) December 31, 2017.

**Table 1:** Characteristics of BP-exposed women by race/ethnicity group, 2010-2017.

	Race/ethnicity group			
Characteristic	White, *N* (%)	Asian, *N* (%)	Other, *N* (%)	Total, *N* (%)
**Number of women**	97 519	25 535	60 556	183 610
**Atypical femur fracture**	83 (0.09)	114 (0.45)	45 (0.07)	242 (0.13)
**Age group, yr**				
**50–64**	29 515 (30.3)	11 204 (43.9)	21 071 (34.8)	61 790 (33.7)
**65–74**	30 836 (31.6)	9127 (35.7)	22 260 (36.8)	62 223 (33.9)
**75–84**	25 665 (26.3)	4121 (16.1)	13 246 (21.9)	43 032 (24.4)
**85+**	11 503 (11.8)	1083 (4.2)	3979 (6.6)	16 565 (9.0)
**EVS, 15-min intervals, median (IQR)**	4.1 (1.1, 9.0)	6.0 (2.7, 10.6)	3.8 (1.2, 7.7)	4.3 (1.3, 8.8)
**BP use duration, yr**				
**<0.25**	55 727 (57.1)	13 732 (53.8)	37 027 (61.2)	106 486 (58.0)
**0.25–<3**	32 412 (33.2)	9623 (37.7)	20 632 (34.1)	62 667 (34.1)
**3–<5**	5868 (6.0)	1546 (6.1)	2076 (3.4)	9490 (5.2)
**5–<8**	2863 (2.9)	568 (2.2)	728 (1.2)	4159 (2.3)
**8**	649 (0.7)	66 (0.3)	93 (0.2)	808 (0.4)
**BP use recency, yr**				
**≤0.25**	73 397 (75.3)	19 644 (76.9)	44 832 (74.0)	137 873 (75.1)
**>0.25–1.25**	14 275 (14.6)	4053 (15.9)	10 564 (17.5)	28 892 (15.7)
**>1.25–4**	6784 (7.0)	1319 (5.2)	3571 (5.9)	11 674 (6.4)
**>4**	3063 (3.1)	519 (2.0)	1589 (2.6)	5171 (2.8)

Data were collected from the KPSC electronic health record (EHR). “Exercise vital signs” (EVS), validated measures of self-reported exercise^12–14^ collected as part of routine care at outpatient visits beginning in 2009, involve 2 questions: (1) “On average, how many days per week do you engage in moderate to strenuous exercise (like a brisk walk)?” and (2) “On average, how many minutes per day do you engage in exercise at this level?” The product of these responses is recorded in the EHR. For each patient in our cohort, we calculated the average minutes/week from the index date through the end of follow-up. This value was transformed to represent the number of weekly 15-min blocks of time spent exercising. Patients with no recorded EVS measures were included in the category of patients reporting no exercise.

Women who sustained AFFs during follow-up were identified via expert review of radiographs of candidate femur fractures identified by ICD-9/10 codes,^11^ and adjudicated using the 2014 ASBMR Task Force AFF case definition.^15^

Age in years, calculated from birth date and index date, was categorized as 50-64, 65-74, 75-84, and 85+. Race/ethnicity was categorized as non-Hispanic White (White), Asian/Pacific Islander (Asian), or Other/Unknown (Other) to align with our previous work with this cohort.^11^ Cumulative duration of BP exposure and time since last use (recency) were determined from KPSC pharmacy records.

This study was approved by the KPSC IRB and conducted under a waiver of consent.

### Statistical analyses

Bivariate associations between race/ethnicity and EVS, age, cumulative duration of BP exposure, and recency of BP use were estimated with a chi-square test for categorical data. Poisson regression models using robust SE and the log of follow-up time as an offset to account for the varying follow-up times of individuals in the cohort were used to estimate associations of patient characteristics with AFF as follows: (1) unadjusted model for race/ethnicity and AFF; (2) unadjusted model for EVS and AFF; (3) Adjusted Model 1—risk of AFF adjusted for both EVS and race/ethnicity; (4) Adjusted Model 2—risk of AFF adjusted for race/ethnicity, age, BP use duration, BP use recency in a single model (this closely replicated our previously published estimates^11^); and (5) Adjusted Model 3—risk of AFF, adding EVS to Adjusted Model 2. Associations are reported as incident rate ratios (IRR) and 95% CI. Analyses were conducted using Stata Statistical Software (Release 18; StataCorp 2023)^16^ and R Statistical Software (Version 4.4.1; R Core Team 2023).^17^

## Results

The cohort included 183 610 BP-exposed women with a median follow-up time of 7.9 yr (IQR 4.8, 7.9) and median age of 69.3 yr (IQR 62.5, 77.7). Of these women, 5503 (3.0%) had no EVS measures during the study period. The cohort was mostly non-Hispanic white (53.1%), Asian or Pacific Islander (13.9%), and other (eg, Hispanic, non-Hispanic Black) or unknown race/ethnicity (33.0%) (Table 1). Many women (*n* = 106 486, 58.0%) had <3 mo of cumulative BP use (~1 prescription dispensed); another 62 667 (34.1%) used BP between 3 mo and <3 yr (Table 1). The overall median number of 15-min blocks of weekly exercise reported was 4.3 (IQR 1.3-8.8) (equal to ~65 min per week).

The association between EVS and race/ethnicity was statistically significant (*p* < .001), with Asian women reporting higher exercise levels. In the model of AFF risk including both EVS and race/ethnicity, the IRR for Asian women (compared to White women) was 5.20 (CI, 3.91-6.90), which was very similar to the unadjusted association between AFF and race/ethnicity (IRR for Asian women 5.12, CI, 3.86-6.79) (Table 2). After adjusting for age group, BP use duration, and BP use recency, the EVS-adjusted IRR for Asian women was 5.03 (CI, 3.77-6.71). In this fully adjusted model, the IRR for each 15-min increase in exercise levels was 0.98 (CI, 0.96-1.00) (Table 2).

**Table 2:** Modeling results evaluating the association of race/ethnicity and EVS with AFF.

	Bivariate associations IRR (95% CI)	Adjusted model 1^a^ IRR (95% CI)	Adjusted model 2^b^ IRR (95% CI)	Adjusted model 3^c^ IRR (95% CI)
**Race/ethnicity**				
**White**	1.00	1.00	1.00	1.00
**Asian**	5.12 (3.86, 6.79)	5.20 (3.91, 6.90)	4.96 (3.72, 6.61)	5.03 (3.77, 6.71)
**Other**	0.90 (0.62, 1.29)	0.89 (0.62, 1.28)	1.05 (0.73, 1.52)	1.03 (0.71, 1.48)
**EVS, 15-min blocks**	1.00 (0.98, 1.02)	0.99 (0.97, 1.01)	—	0.98 (0.96, 1.00)
**Age group, yr**				
**50–64**			1.00	1.00
**65–74**			1.74 (1.27, 2.38)	1.72 (1.25, 2.36)
**75–84**			1.67 (1.17, 2.38)	1.57 (1.10, 2.26)
**85+**			0.34 (0.12, 0.94)	0.31 (0.11, 0.85)
**BP use duration, yr**				
**<0.25**			1.00	1.00
**0.25–<3**			3.06 (2.22, 4.22)	3.10 (2.25, 4.28)
**3–<5**			8.00 (5.50, 11.60)	8.20 (5.64, 11.90)
**5–<8**			8.75 (5.36, 14.30)	8.92 (5.46, 14.60)
**8+**			17.00 (7.72, 37.40)	17.40 (7.89, 38.20)
**BP use recency, yr**				
**≤0.25**			1.00	1.00
**>0.25–1.25**			0.49 (0.33, 0.75)	0.49 (0.33, 0.75)
**>1.25–4**			0.55 (0.30, 1.01)	0.54 (0.29, 1.00)
**>4**			0.15 (0.02, 1.08)	0.15 (0.02, 1.06)

^a^Adjusted model 1 included both race/ethnicity and EVS.

^b^Adjusted model 2 approximates a previously published model, which yielded a 5-fold increase in the risk of AFF for Asian women compared to White women.^11^

^c^Adjusted model 3 adds EVS to the variables included in adjusted model 2.

Abbreviations: AFF, atypical femur fractures; BP, bisphosphonate; EVS, exercise vital sign; IRR, incidence rate ratios.

## Discussion

Contrary to our hypothesis, our findings suggest that increasing levels of moderate to strenuous activity are not associated with increased risk for AFF. Rather, in the fully adjusted model (Adjusted Model 3), weekly exercise was not associated with the risk of AFF. In addition, the inclusion of EVS in this fully adjusted model did not substantially attenuate the 5-fold increased AFF risk among Asian women previously reported by our group^11^ and other researchers.^1–5^

Strengths of our study include the large population of BP-exposed women with long duration of follow-up and radiographically adjudicated AFFs, for which self-reported exercise information was routinely collected as part of clinical care. These data were further enriched with longitudinal pharmacy data enabling estimation of total duration of BP exposure and recency of exposure.

In conclusion, despite the inclusion of novel measures of weekly exercise levels in our models, we do not observe any attenuation of the strong association of race/ethnicity with the incidence of AFF, suggesting that the association of EVS with race/ethnicity is insufficient to account for the magnitude of the association of Asian race with AFF.

## Data Availability

The data underlying this article may be shared upon reasonable request to the site corresponding author.
